# The neurobiological cravings signature (NCS) as a predictive neuromarker of clinical outcomes in alcohol use disorder

**DOI:** 10.1038/s41386-026-02369-3

**Published:** 2026-02-25

**Authors:** Andreas Löfberg, Nicholas Harp, Irene Perini, Robin Kämpe, Hanna Karlsson, Michal Pietrzak, Hedy Kober, Markus Heilig

**Affiliations:** 1 Center for Social and Affective Neuroscience, Department of Biomedical and Clinical Sciences, Linköping University, Linköping, Sweden; 2 Department of Psychiatry, Linköping University Hospital, Linköping, Sweden; 3 Department of Psychology, University of California, Berkely, CA USA

**Keywords:** Addiction, Predictive markers

## Abstract

The Neural Craving Signature (NCS), a machine learning derived neuroimaging biomarker, differentiates individuals with from those without substance use disorders (SUDs), but has not been evaluated for predicting clinical outcomes. In a secondary analysis, we applied the NCS to fMRI cue-reactivity data from 39 participants in a published, negative RCT of repetitive transcranial magnetic stimulation (rTMS) for Alcohol Use Disorder (AUD). NCS scores predicted craving [Penn Alcohol Craving Scale (PACS)], both at the time of fMRI (*R* ^2^ = 0.29, 95%, CI [0.27, 0.73], t(36) = 3.86, *p* = 0.0005), and during repeated study visits (β = 4.6, SE = 5.3, t(39.15) = 1.17, *p* < 0.0001). NCS also classified AUD severity (Addiction Severity Index, ASI, alcohol subscale—β = 0.14, *SE* = 0.04, *p* = 0.0016, *R²* = 0.24; Alcohol Use Disorder Identification Test, AUDIT, β = 5.32,*SE* = 1.46, *p* < 0.0025, *R²* = 0.22). Most importantly, the NCS predicted alcohol use, both measured by self-reported percent heavy drinking days (HDD%; β = 10.19, SE = 4.46, t(38.23) = 2.28, *p* = 0.028) and the biomarker phosphatidyl ethanol (PEth; β = 0.32, SE = 0.15, t(37.10) = 2.15, *p* = 0.038). Participants with below median NCS scores had a lower likelihood of relapse than those above median (Cox regression—HR = 0.35, 95% CI [0.16–0.80], *p *= 0.013). NCS identified relapse cases with an area under the curve of 0.79 (SE = 0.077, z = 3.8, *p* = 0.0001), achieving 66.7% sensitivity and 77.8% specificity at optimal NCS score. These findings provide initial support for the NCS as a predictor of clinical outcomes in AUD.

## Introduction

Alcohol use disorder (AUD) is a chronic-relapsing condition characterized by craving for alcohol, and continued use despite negative consequences. The prevalence of AUD is ~10% in Western countries, yet fewer than 10% of affected individuals receive evidence-based care [1, 2]. For those who are treated, outcomes are highly variable, and the modal outcome is often relapse [3]. This variability underscores a need for biomarkers to guide interventions [4], but despite recent developments of neuroimaging biomarkers, there is a lack of robust predictors of treatment outcomes [5, 6]. Importantly, brain-based biomarkers can overcome limitations of self-reports (e.g., social desirability biases; [7]), limited self-insight among those struggling with substance use disorders [8, 9], and move the field closer to personalized interventions [4].

A recent meta-analysis demonstrated that craving—including cue-induced craving—consistently predicts future substance use and relapse [10], which held true for both real (in vivo) and image (pictorial) drug cue presentations. These findings suggest that neural processes underlying craving and cue reactivity may have clinical relevance as predictors of drug use and treatment outcomes. Multiple functional magnetic resonance imaging (fMRI) paradigms have been established to collect objective measures of the neural responses to craving-provoking stimuli, e.g., images, stress, or priming doses; [11, 12]. Using these types of stimuli, a growing number of studies has attempted to link neural activity in response to craving provoking stimuli, such as alcohol-associated cues or stressors, and clinical outcomes in AUD, e.g., images, stress; [11–13]. However, these efforts did not meet validation standards required of clinical biomarkers [6, 14]. External validity has also been limited by the use of regions of interest (ROIs) defined post-hoc [ROI; [15].

Recent advances in fMRI techniques and machine learning demonstrated that affective experiences such as pain [16], reward [17], and negative affect [18] involve widely distributed patterns of brain activity, rather than isolated regions. Recently, Koban, Wager, and Kober [19] used machine learning and fMRI data to develop the Neurobiological Craving Signature (NCS), a multivariate pattern of brain activity that predicts subjective craving ratings on a trial-by-trial basis, and that successfully classified users versus non-users for a variety of drugs. A key question prompted by these results is whether the NCS is also predictive of future treatment outcomes, in a manner that parallels self-reported craving. In the published literature, the NCS has, as of August 2025, only been applied to one external dataset of AUD patients [20], but this study did not evaluate its ability to predict clinical outcomes.

Here, we evaluated the NCS as a predictive neuromarker of craving, disorder severity, and relapse to alcohol use. We chose these outcomes a priori, based on our earlier work [19]. This was a secondary analysis of participants in a randomized, sham-controlled clinical trial (RCT) that evaluated repetitive transcranial magnetic stimulation (rTMS) with an H-coil configuration (Brainsway), targeting bilateral insula, as a treatment for AUD [21]. Because insula-targeting rTMS did not influence craving or alcohol use during the 3-month follow up phase, we could pool data across conditions (while nevertheless controlling for treatment allocation). We applied the NCS to post-treatment fMRI alcohol cue reactivity task data, and hypothesized that participants with high NCS expression would relapse to heavy alcohol use at a higher rate than participants with low NCS expression. We also examined whether NCS score would predict subjective cravings and AUD severity.

## Method

### Overview of the clinical trial

In brief, the RCT evaluated rTMS targeting the insula bilaterally as a potential treatment for AUD at Linköping University, Sweden. The trial was pre-registered on ClinicalTrials.gov (NCT02643264) and approved by the LiU Regional Ethics Committee. Full methodological details are available in Perini et al. [21].

### Participants

Fifty-six participants with DSM-IV alcohol dependence ≈moderate-severe DSM-5 AUD, see [Diagnostic and Statistical Manual of Mental Disorders, fourth edition; [22], this is equivalent to ≈moderate-severe DSM-5 AUD, see [23]] were enrolled September 2015–October 2018. Eligibility criteria were: (i) current alcohol dependence; (ii) recent alcohol use; and (iii) 25–64 years of age. Exclusion criteria included (i) more than mild cognitive impairment by Mini Mental State Examination MMSE < 24; [24]; (ii) schizophrenia, bipolar, or other psychotic disorder; (iii) any clinically significant neurological disorder or lesion; (iv) hearing impairment; or (v) pregnancy. All participants provided written informed consent.

### Assessments and treatment

Baseline assessments included the Alcohol Use Disorder Identification Test AUDIT; [25], the Alcohol Dependence Scale ADS; [26], and the Addiction Severity Index ASI; [27, 28]. All participants underwent a clinician-administered psychiatric evaluation using the Structured Clinical Interview for DSM-IV diagnosis SCID-CV; [29]. Severity of depression and anxiety symptoms was assessed using the self-report version of the Comprehensive Psychopathological Self-Rating Scale for Affective Symptoms CPRS-SA; [30] and the Clinical Global Impression CGI; [31]. Participants received 15 once-daily 20 min sessions of rTMS (real or sham) Monday-Friday over three weeks, followed by a post-treatment fMRI with an alcohol cue reactivity task (see below for details). They returned for follow-up visits at 1-, 2-, 4-, 8-, and 12-weeks post-treatment. rTMS was delivered using a Magstim Rapid stimulator equipped with an H8 coil (Brainsway) to target insula bilaterally. Treatment intensity was set at 120% of the individual motor threshold (50 x 3 s trains at 10 Hz, with 20 s inter-train intervals, for a total of 1500 pulses/session), while sham stimulation mimicked the sensory experience of active treatment without significant cortical penetration. Immediately prior to each rTMS session, participants briefly handled an alcoholic beverage to heighten craving.

### fMRI

#### Data acquisition

fMRI data were acquired on a Philips Ingenia 3 T scanner (Philips Healthcare, Best, The Netherlands) with a 32-channel head coil. Blood oxygen-level-dependent (BOLD) data were acquired at TR = 2000ms; TE = 30 ms; resolution = 3.4 × 3.4 × 4 mm; no slice gap; 140 volumes per run. Anatomical data were collected with a high-resolution 3D T1-weighted Turbo Field Echo (TFE) scan at TR = 7.0 ms; TE = 3.2 ms; voxel resolution = 1 × 1 × 1 mm; no slice gap.

### Cue-Reactivity task

**Fig. 1:**
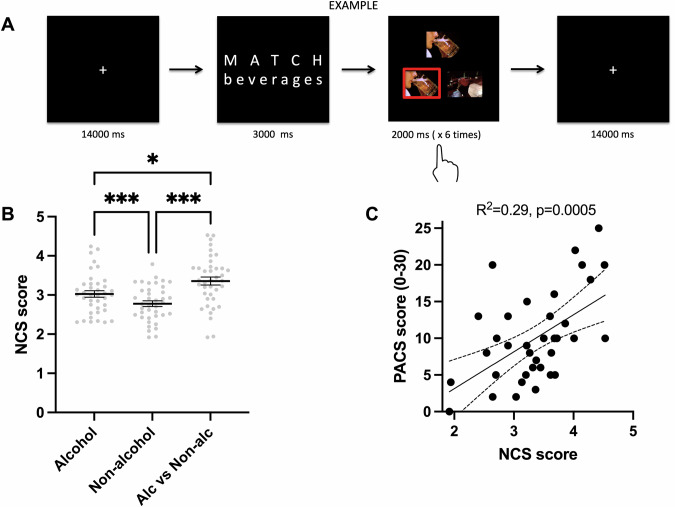
fMRI cue-reactivity task, NCS scores by condition, and craving prediction. **A** Outline of the beverage-matching cue-reactivity task used to evoke craving and associated neural responses. Participants were instructed to match pictures of alcoholic and non-alcoholic beverages, or, as control for attention- and motor-related processes, geometric shapes. **B** NCS scores by fMRI cue type. Thick lines are mean values ± SE error bars, with individual datapoints in light gray. P-values from paired *t* tests (*0.05, **0.005, *** 0.001). **C** Scatter plot showing NCS scores applied to the alcohol vs. non-alcohol contrast image on the X-axis, and PACS scores at the fMRI visit on the Y-axis. Dotted line representing Pearson correlation of NCS scores and PACS scores, dotted lines indicate 95% CI Confidence Intervals.

Alcohol cue reactivity was tested using a paradigm modified from a widely used affective picture matching task [32]. This paradigm differs from passive viewing, in that the requirement for a matching response is thought to ensure that participants attend to the stimuli (Fig. 1A). On every trial, participants saw an instruction screen for 3000 ms, noting “match beverages” or, on control trials, “match shapes.” On “match beverages” trials, participants were shown a “target” image of alcoholic or non-alcoholic beverages on top, and two “choice” images of alcoholic or non-alcoholic beverages on the bottom, and were asked to press a button (right hand) to match the target with the correct choice. Shape-matching was used to control for processes unrelated to the nature of the stimuli, such as attention and motor action. The beverage images from [33] were matched in terms of valence and arousal ratings as well as objective indices (e.g., brightness). For two runs, each category (i.e., alcohol, non-alcohol, or shapes) appeared in three blocks in a random order, each block comprised six consecutive 2-second trials. Each block was followed by a fixed 14-second inter-block interval.

### Preprocessing

Data were preprocessed using a standard fMRIPrep workflow 24.1.1; [34], which is based on Nipype [35], with default settings to enhance reproducibility. T1‑weighted images underwent intensity correction (N4BiasFieldCorrection, ANTs), skull‑stripping (antsBrainExtraction), tissue segmentation (FAST, FSL), and nonlinear normalization to MNI152NLin2009cAsym (ANTs). The BOLD timeseries for both runs were realigned (MCFLIRT, FSL) and co‑registered to each participant’s T1w image using boundary‑based registration. Motion regressors were extracted for nuisance covariates in first level analysis. Images were smoothed with a 6 mm FWHM kernel using SPM12 (Wellcome Department of Cognitive Neurology, London, UK) implemented in Matlab 2024b (version: 24.1, Natick, Massachusetts, USA).

### fMRI GLM

Subject-level data were modeled in SPM12 using custom scripts [https://github.com/canlab]; [19]. Three regressors of interest were modeled in the analysis capturing the 2-second trials of each image category (i.e., shape, alcohol, non-alcohol). Regressors of no interest included instructions, button presses, 24 movement regressors (i.e., three rotation and three translation parameters, their derivatives, their squares, and derivatives of their squares) and spike regressors (i.e., global outliers). One beta image was generated for each regressor at each trial run. Alcohol and non-alcohol betas from both runs were then used to create one single alcohol vs. non-alcohol contrast image for the session.

### NCS application

The primary application of the NCS pattern was to the alcohol, non-alcohol, and alcohol vs. non-alcohol contrast images using custom MATLAB scripts, which generated a continuous numeric score representing the dot (scalar) product of the contrast image and the whole-brain NCS pattern (i.e., NCS score; Fig. 1B). The NCS score was used as predictor in subsequent analyses. For survival analysis, the cohort was split by the group median NCS score value, with individuals above the threshold classified as “High NCS” and those below as “Low NCS.”

### Outcomes

The primary outcomes were alcohol use and craving over 15 weeks (treatment and follow-up), and time to relapse to heavy drinking following completion of treatment. The preregistered definition of heavy drinking day was ≥5 standard drinks of 12 g alcohol ( > 60 g alcohol) in a day, in accordance to the Swedish National Board of Health and Welfare [36]. Alcohol use was assessed with Timeline Follow Back [TLFB]; [37] and presented as percentage of heavy drinking days per week (HDD%), and craving with the Penn Alcohol Craving Scale [PACS]; [38], collected at each visit. Phosphatidyl ethanol [PEth]; [39] was used as an objective, quantitative blood-based biomarker of alcohol use. PEth has excellent specificity, sensitivity, and quantitative properties [40]. Concentrations <0.05 μmol/L indicate abstinence; 0.05–0.30 indicate moderate use; and levels >0.3 reflect heavy alcohol use.

### Statistical analysis

We conducted statistical analyses in R (v4.4.1; R Core Team, 2024). We used *t* tests, Fisher’s Exact Test, Pearson correlations, and linear models for pairwise comparisons. We assessed relationships between NCS scores and repeated measures of craving (PACS) and alcohol use (PEth, HDD%) with linear mixed-effects models, including fixed effects for NCS scores, time in weeks from treatment start, as well as treatment condition, age, and sex as covariates. The models included random intercepts for subject. We then compared models with fixed and random slopes for time using a likelihood ratio test. To evaluate significance of the linear mixed-effects models in R, maximum likelihood estimation was employed with Satterthwaite’s approximation for degrees of freedom, which is appropriate for the sample size of 39 [41]. The random-effects correlation structure was an unstructured (full) variance–covariance matrix. Likelihood ratio test was used for model comparison.

We conducted survival analyses using Kaplan–Meier plots and Cox regression, with estimates of hazard ratios and corresponding p-values. Schoenfeld residuals were used to test the assumptions of the model. Predictors included sex, age, treatment condition, and NCS group (high vs low). Because the proportional hazards assumption of the Cox regression was borderline significant, we also carried out a sensitivity analysis in which we compared relapse-free survival between the groups using Restricted Mean Survival Time (RMST) as an alternative estimand that does not rely on the proportional hazard assumption. The RMST represents the average time to relapse during the 12 week-long follow-up. We estimated RMST differences between the High vs Low NCS group, with age, sex, and treatment as covariates.

NCS classification performance (i.e., identification of relapse cases) was assessed by Receiver Operating Characteristic (ROC) plots. The ROC analysis for was performed using custom scripts in MATLAB (https://github.com/canlab). The area under the curve (AUC) was calculated and interpreted according to Mandrekar’s [42] guidelines: 0.5–0.7 indicates low accuracy, 0.7–0.8 moderate, 0.8–0.9 excellent, and >0.9 outstanding. The optimal cutoff threshold was determined by the Index of Union (IU), defined as the point at which sensitivity and specificity jointly deviate the least from the AUC [IU(c)=|Sensitivity(c)–AUC | +|Specificity(c)–AUC | ]; [43]. Positive and negative predictive values were also computed.

As a complement to our statistical approach, we also conducted Bayesian survival modeling. Such modeling provides full posterior distributions for hazard ratios [44]. We ran the Bayesian Cox model to complement the frequentist Cox model, to estimate the uncertainty about whether low NCS is protective against relapse. We computed a Bayes Factor comparing a full model (including NCS group as predictor of relapse) against a null model (excluding NCS, only including time, sex, age, and treatment) to assess the strength of evidence in favor of a protective effect of low NCS. Fitting a Bayesian logistic regression and deriving ROC curves from its posterior predictive probabilities yields a full distribution of parameters, thus offering a richer assessment of discriminative performance and quantifying uncertainty beyond single-point frequentist estimates.

We conducted Bayesian analyses using the R package brms, which utilizes Stan’s Hamiltonian Monte Carlo sampling to generate posterior distributions via Markov Chain Monte Carlo (MCMC). First, a Weibull survival model was fit for time-to-relapse, yielding a hazard ratio distribution. Second, we assessed the discriminative performance of NCS expression by fitting a Bayesian logistic regression model with relapse as the binary outcome. From the posterior predicted probabilities, we derived the ROC curve and computed the AUC for each posterior draw, yielding a full distribution of AUC values. Both models included covariates for NCS group, treatment condition, age, and sex. For the survival model, we set weakly informative priors for most regression coefficients. For the NCS group effect, we used a more informative prior, HR = 0.2 for Low NCS. This reflects the more conservative estimates from two prior studies that have attempted to use fMRI regions to predict relapse in AUD with hazard ratios ranging from 0.12 to 0.20 [45, 46], implying a protective effect for low NCS scores. The Weibull shape parameter was assigned a Gamma(1, 1) prior. In the logistic regression analysis, which models the binary relapse outcome with a Bernoulli likelihood and logit link, default weakly informative priors were employed. Four MCMC chains were run with 5,000 iterations each (including 2,000 warm-up iterations). Posterior predictive checks were performed, and convergence was verified via Rhat values and effective sample sizes.

## Results

### Participant characteristics

A CONSORT-graph of participant disposition is provided in Supplementary Fig. S1. Of 56 participants enrolled, 43 completed the treatment phase. The first 4 fMRI scans were excluded due to the use of different fMRI scan parameters, for a final sample of *N* = 39 participants with complete data. Baseline characteristics, overall as well as stratified by median NCS expression (median=3.37), are presented in Table 1. Groups were balanced across treatment condition (rTMS vs. sham), age, and sex (all ps>0.10), as well as baseline assessments. Only the ASI alcohol problem severity subscale differed between the groups, where the high NCS group scored significantly higher than the low group (unpaired t(32.99) = 3.33, *p* = 0.002).

**Table 1:** Baseline characteristics of the study participants.

Variable (Mean ± SD)	Full cohort	High NCS	Low NCS	*p* value
Number of participants (n female)	39 (7)	20 (5)	19 (2)	0.41
Treatment rTMS (n Sham)	19 (20)	7 (13)	12 (7)	0.11
Age	52.23 ± 9.49	52.80 ± 7.63	51.63 ± 11.31	0.71
Relapse cases (no relapse)	30 (9)	19 (1)	11 (8)	0.008
Number of AUD Dependence criteria (DSM-IV)	5.92 ± 1.18	5.70 ± 1.08	6.16 ± 1.26	0.23
MMSE	28.97 ± 1.04	29.15 ± 0.81	28.79 ± 1.23	0.29
TLFB grams/week	415.72 ± 301.28	492.60 ± 341.62	334.79 ± 234.43	0.1
AUDIT	24.44 ± 7.24	25.65 ± 7.51	23.16 ± 6.91	0.29
ADS	18.36 ± 7.53	16.25 ± 6.93	20.58 ± 7.68	0.07
CPRS-SA				
Depression	7.92 ± 5.09	8.07 ± 5.50	7.76 ± 4.76	0.85
Anxiety	6.92 ± 4.68	6.62 ± 5.02	7.24 ± 4.42	0.69
Number of smokers/total n	28/39	14/20	14/19	0.99
ASI				
Medical Problems	0.29 ± 0.31	0.30 ± 0.32	0.27 ± 0.30	0.82
Psych Problems	0.23 ± 0.19	0.23 ± 0.19	0.23 ± 0.20	0.98
Family/Social Problems	0.24 ± 0.18	0.22 ± 0.22	0.27 ± 0.14	0.37
Alcohol Problems	0.58 ± 0.17	0.65 ± 0.16	0.49 ± 0.13	0.002
Drug Problems	0.05 ± 0.06	0.05 ± 0.06	0.04 ± 0.08	0.75
Legal Problems	0.05 ± 0.07	0.04 ± 0.13	0.06 ± 0.21	0.72
Framewise displacement	0.29(0.16)	0.25(0.09)	0.33(0.20)	0.11
Outlier volumes	4.05(3.29)	3.90(3.34)	4.21(3.33)	0.77

*P* values derived from *t* tests for continuous variables and Fisher’s exact test for categorical (i.e., proportion male/female, treatment allocation, and relapse).

*rTMS* repetitive transcranial magnetic stimulation, *MMSE* Mini Mental State Exam, *TLFB* Time Line Follow Back, *AUDIT* Alcohol Use Disorder Identification Test, *CPRS-SA* Comprehensive Psychopathological Self-Rating Scale for Affective Symptoms, *ADS* Alcohol Dependence Severity, *ASI* Addiction Severity Index.

### Cue reactivity

NCS scores derived from the alcohol and non-alcohol cue contrasts, respectively are shown in Fig. 1B. NCS scores were higher for alcohol cues (mean ± SD: 3.03 ± 0.52) than non-alcohol cues (2.78 ± 0.46) in the full cohort (paired t(38) = 4.07, *p* = 0.0002). The NCS scores derived from the alcohol-vs.-non-alcohol contrast had a mean of 3.36 ( ± 0.64), significantly higher than NCS scores from both the alcohol (paired t(38) = –2.8, p = 0.008) and non-alcohol beta images (paired t(38) = –4.03, *p* = 0.0002).

### Predicting craving at fMRI scan

We performed a multiple linear regression to examine whether NCS scores from the alcohol-vs.-non-alcohol contrast predicted concurrent craving scores (PACS) from the day of the fMRI scan. The overall regression model was significant, both with (*F*(3,34) = 6.61, *p* = 0.001; adjusted *R*² = 0.31) and without sex and age as covariates (*F*(1,36) = 14.88, *p* = 0.0005; adjusted *R*² = 0.29; Fig. 1C). Higher NCS significantly predicted greater PACS scores (β = 4.97, SE = 1.30, *t* = 3.84, *p* < 0.001), consistent with the original NCS findings [19]. Sex was not significantly associated with PACS (*p* = 0.79), and higher age showed a marginal association with lower PACS scores (β = -0.18, SE = 0.09, *t* = -2.02, *p* = 0.052).

### NCS and AUD severity

**Fig. 2:**
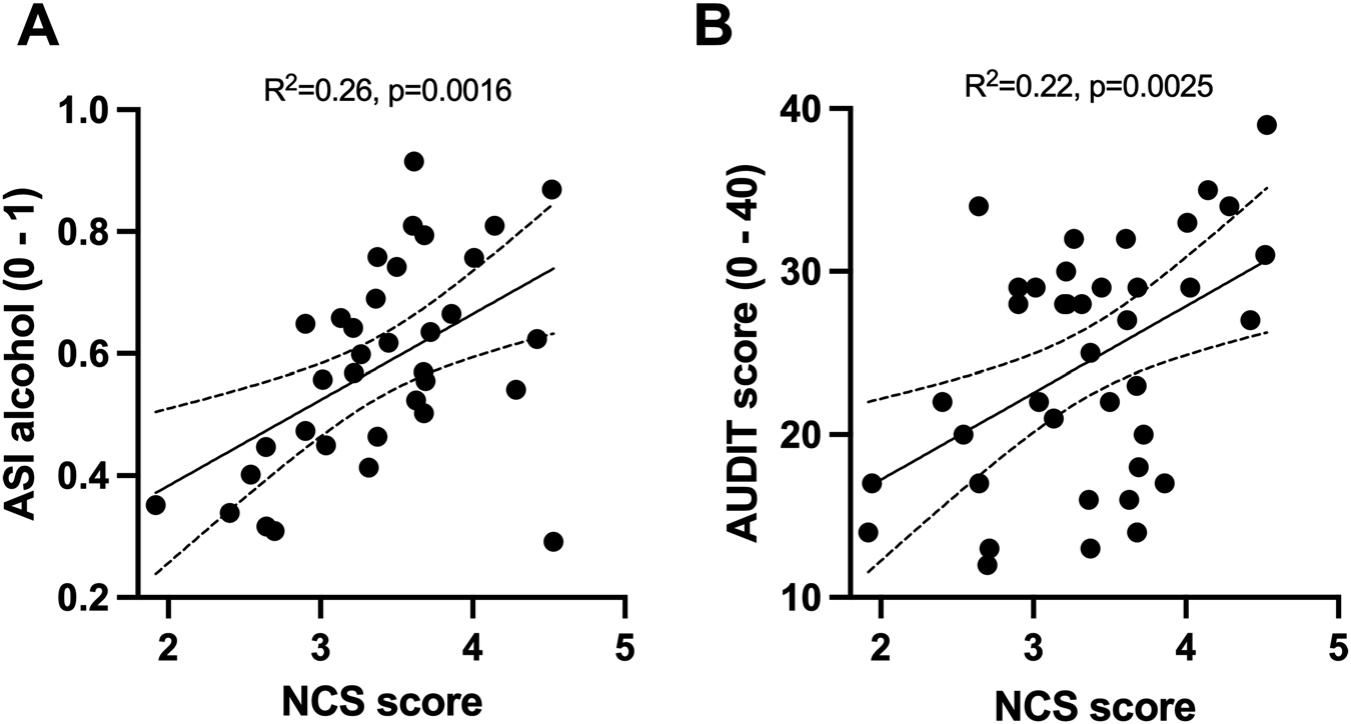
Classification of AUD severity based on NCS scores to alcohol vs non-alcohol cues. **A** Scatter plot showing NCS from alcohol vs non-alcohol contrasts on the X-axis, baseline ASI alcohol problem severity subscale score on Y. **B** Scatter plot showing NCS from alcohol vs non-alcohol contrasts on the X-axis, baseline AUDIT scores Y. Solid lines represent Pearson correlation, dashed lines indicate 95% CI. AUDIT Alcohol Use Disorder Identification Test, ASI Addiction Severity Index, alcohol subscale.

We assessed the relationship between NCS and AUD severity as an extension of the classification of drug users vs non-users from the original NCS publication. A simple linear regression showed a positive association between NCS scores from the alcohol-vs.-non-alcohol contrast and the ASI alcohol problem severity subscale scores (β = 0.14, SE = 0.04, F(1, 33) = 11.82, *p* = 0.0016), explaining approximately 26.4% of the variance (adjusted R² = 0.24). The model was further strengthened when controlling for sex and age (β = 0.13, SE = 0.04, F(3, 31) = 5.25, *p *= 0.0038, adjusted R² = 0.34, Fig. 2A). The NCS scores were also associated with AUDIT scores at baseline, which increased by 5.3 points for each one-unit increase in NCS (β = 5.3, SE = 1.6, F(1,37) = 10.48, *p* < 0.0025, adjusted R² = 0.22, Fig. 2B). The model improved to explain 37% variance after adding sex and age as covariates (β = 5.65, SE = 1.55, F(3,35) = 6.97, *p* < 0.00084, adjusted R² = 0.37). However, the NCS scores were not correlated to ADS scores (*p* = 0.5, adjusted R² = 0.007).

### Prediction of craving and alcohol use

**Fig. 3:**
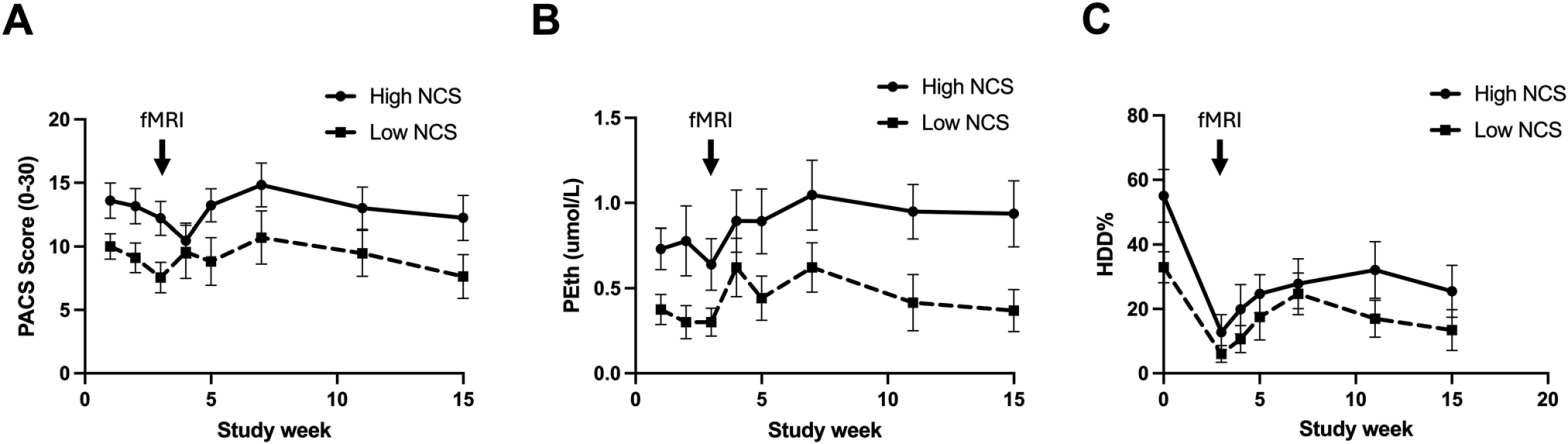
NCS, craving, and drinking over time. Graphs are showing (**A**) mean PACS scores; (**B**) PEth and (**C**) HDD% values by visit, for the two participant groups, defined by a median spit by NCS scores from the alcohol vs non-alcohol contrasts: a high NCS group (above median, solid line) and a low NCS group (below median, dashed line). Values are means ± SE. fMRI scan session at the end of treatment week 3 marked by the arrow. HDD% = percentage heavy drinking days ( > 60 g alcohol/day), averaged since last study visit.

For repeated measures over 15 weeks (3 weeks of treatment, 12 weeks follow-up), we employed linear mixed-effects models to examine the relationships between NCS scores from the alcohol-vs.-non-alcohol contrast, PACS scores (Fig. 3A), PEth values (Fig. 3B), and %HDD (Fig. 3C) for each participant. The likelihood ratio test indicated that the inclusion of random slopes significantly improved model fit both for PACS (χ²(2) = 24.89, *p* < 0.001) and PEth models (χ²(2) = 22.16, *p* < 0.001), but only marginally so for HDD% (χ²(2) = 5.27, *p* < 0.07). These finding demonstrates considerable variability among individuals in how PACS and PEth scores changed over time.

Replicating and extending prior work [19], we found that NCS scores from the alcohol-vs.-non-alcohol contrast predicted craving over time, measured via PACS (β = 4.68, *SE* = 1.02, *t*(38.42) = 4.58, *p* < 0.0001), indicating that for each unit increase in NCS expression, PACS scores increased by approximately 4.7 points. In this model, age had a modest but significant negative effect on PACS scores (β = –0.24, *p* = 0.001) but time (p = 0.99) and sex (*p* = 0.12) had not. The intercept for PACS was estimated at 5.60, representing the baseline PACS score when all other variables are zero.

The NCS scores from the alcohol-vs.-non-alcohol contrast further predicted alcohol use over time, both via the objective biomarker PEth, and by self-report. Each unit increase in NCS increased PEth by approximately 0.32 units (β = 0.32, SE = 0.15, t(37.10) = 2.15, *p* = 0.038). The intercept for PEth was estimated at 1.14. Similarly, the NCS predicted self-reported heavy drinking days, measured via percent heavy drinking days, calculated from the TLFB (HDD%; β = 9.89, SE = 4.48, t(38.25) = 2.2, *p* = 0.033). Neither time nor sex had any significant effects on PEth or HDD% (all *p* ≈ 0.5).

### Relapse prediction

**Fig. 4:**
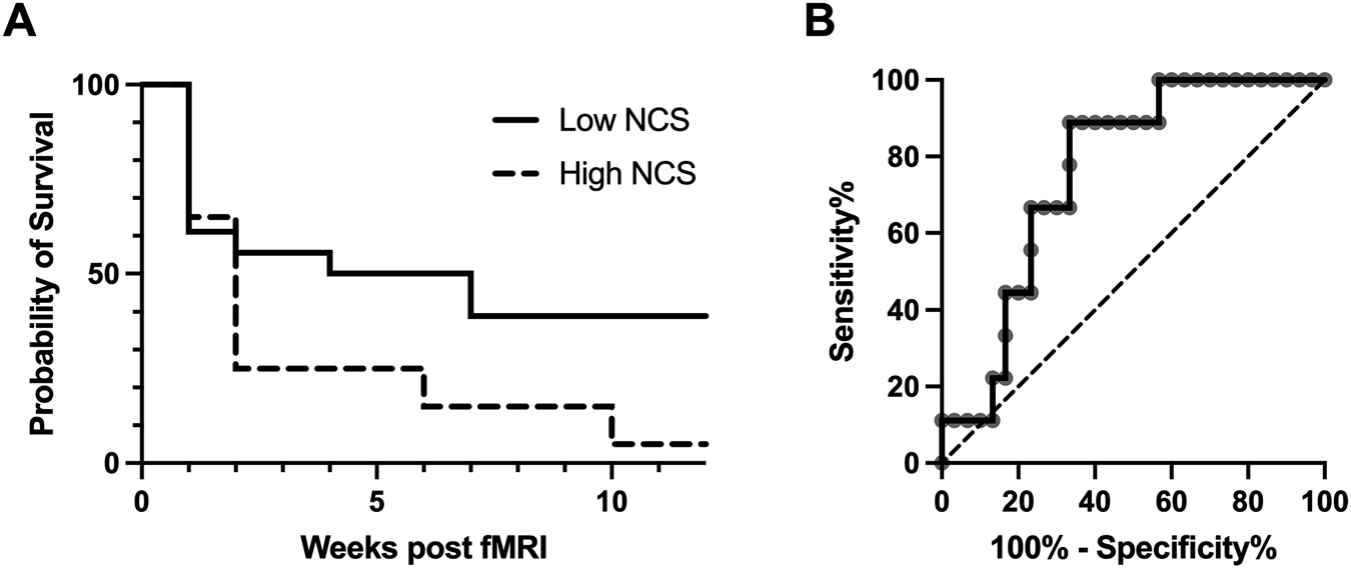
NCS scores from the alcohol vs non-alcohol contrast as predictor of survival to relapse to heavy alcohol use. **A** Kaplan-Meyer plot of time to relapse to heavy alcohol use ( > 60 g/day) during the 12 weeks following the post-treatment fMRI session, stratified by NCS group (High NCS: >3.37, dashed line, *n* = 20; Low NCS: ≤3.37, solid line, *n* = 19). All NCS scores are from the alcohol vs non-alcohol contrasts. **B** ROC analysis showing the discriminative performance of NCS applied to the alcohol vs non-alcohol contrast to identify cases of relapse. Dashed diagonal line representing AUC of 0.5 for reference.

We found that the NCS predicted relapse across several analyses. Survival to relapse, stratified by median NCS (High:>3.37, *n* = 19; Low:≤3.37, *n* = 20), is shown in a Kaplan-Meier plot in Fig. 4A, where the High NCS group relapsed faster. Cox regression confirmed that participants below median NCS score from the alcohol-vs.-non-alcohol contrast had a significantly lower hazard of relapse (HR = 0.35, 95% Confidence Interval, CI [0.16–0.80], *p* = 0.013). In contrast, neither treatment (HR = 0.73, *p* = 0.43), age (HR = 0.97 per year, *p* = 0.14), nor sex (HR = 2.68, p = 0.08) were significant predictors. The overall model fit was statistically significant (likelihood ratio test = 10.39 on 4 df, *p* = 0.03; concordance=0.675). Schoenfeld residuals indicated a borderline proportional hazards assumption for NCS group (*p* = 0.058), with no violations for the other covariates. The effect of NCS on relapse was attenuated when including PACS at the time of the fMRI as a covariate in the Cox regression model (all *p* > 0.1), but we did not conduct formal mediation testing due to the small sample size [47] and challenges associated with conducting mediation in proportional hazard models (e.g., rarity of event occurrence; [48]). We confirmed the robustness of the Cox-regression results both using the non-parametric log-rank test, which does not rely on a proportional hazard assumption (p = 0.023). Similarly, the RMST analysis showed that Low NCS individuals remained relapse-free for 3.97 weeks (95% CI [1.31–6.63], *p* = 0.003) longer than High NCS individuals during the 12- week follow-up. Male sex ([–6.34 to 0.17], *p* = 0.04) was associated with 3.25 weeks shorter, and higher age ([0.02–0.26], *p* = 0.03) with a 1 day longer relapse-free survival per year, while treatment had no effect (*p* = 0.6). The Weibull model yielded a posterior distribution with a coefficient for Low NCS of –1.38 (95% CI [–2.25,–0.59], Fig. S2), indicating a significantly lower hazard of relapse (median hazard ratio = exp(–1.38) ≈ 0.25). Bayes Factor for the Cox model was 4.14, indicating moderate evidence in favor of NCS predicting survival. See plot and full model output in Supplementary Materials.

We also tested whether PACS scores predicted relapse. PACS (week 3) scores, along with age, sex, and treatment covariates, resulted in a non-significant model (likelihood ratio test = 6.94 on 4 df, *p* = 0.10; concordance = 0.655), and the point estimate for PACS was not significant (HR = 1.06, 95% Confidence Interval, CI [1.00–1.13], *p* = 07). There was also no benefit to adding PACS (week 3) to the model with NCS group (reported above), as model comparison was non-significant (*X*^*2*^(1) = 0.35, *p* = 0.56). Thus, there appears to be unique variance associated with the NCS in prediction of relapse, above and beyond craving self-reports.

We further assessed whether the NCS could identify cases of relapse into heavy alcohol use, using a ROC analysis (Fig. 4B). We found that the area under the curve (AUC) was 0.79 (SE = 0.077, z = 3.8, *p* = 0.0001). The optimal cut-off point, determined by IU, was 3.27. At this threshold, NCS reached a sensitivity of 66.7% and a specificity of 77.8%. The Bayesian ROC analysis supported these results, and showed an AUC distribution with a median of 0.91, with a 95% credible interval ranging from 0.84 to 0.92 (Supplementary Fig. S3). This demonstrates excellent discrimination, indicating that the probability of correctly identifying a relapse based on NCS expression is high.

## Discussion

This study is, to our knowledge, the first application of the NCS [19] for predicting clinical outcomes, including relapse to heavy alcohol use in AUD. Specifically, we explored if the NCS score tracked with clinically relevant outcomes, namely alcohol use measured by self-report (TLFB), and an objective biomarker (PEth). We found that NCS scores predicted both. We also validated the ability of NCS to predict self-reported craving, and extended prior results by showing that it is associated to AUD severity.

Addiction is widely considered to be a brain disease [49, 50], but the development of brain-based biomarkers has lagged behind other fields such as Alzheimer’s disease and depression [6]. Our findings show that the NCS may serve as both a prognostic and monitoring (“theragnostic”) neuromarker for AUD [51], advancing established cue-reactivity neuroimaging techniques [12]. The NCS offers an objective complement to self-report measures like the PACS, which are inherently vulnerable to recall errors and social desirability bias [52]. As such, the NCS objectively captures real-time neural responses to alcohol cues and bridges the gap between neural activity and quantifiable biomarkers of alcohol use, such as PEth.

Our findings also bridge two recent advances in the field. First, the role of cravings as a predictor of clinical outcomes was long debated see e.g., [53], but recent meta-analytical findings provided compelling support for outcome prediction by self-reported cravings [10]. Second, although the prediction of craving self-reports by NCS scores has been established [19]—a finding we validate here with a different sample, scanner, cue-reactivity stimuli, and measures—it remained unclear whether the NCS would also predict clinical outcomes. Our findings provide initial support for both propositions, as the NCS predicted alcohol use, assessed both by the clinically established, specific biomarker PEth and by self-report. The strong association between NCS scores and both AUDIT and ASI-alcohol scores indicate that the NCS is sensitive to AUD severity [54–56]. Importantly, the classification analysis on high vs. low ASI alcohol problem severity subscale scores provides a conceptual extension of the ability of NCS to classify between individuals with vs. without SUD [19].

The NCS offers two critical advantages. First, unlike previous approaches using masks focused on individual regions, like the ventral striatum [46] or orbitofrontal cortex [57], the NCS employs a whole-brain multivariate pattern, defined and cross-validated a priori. It better captures distributed neural processes underlying complex phenomena like craving, explaining more variance than single-region approaches, while reducing susceptibility to measurement noise [58, 59]. Second, it avoids circular inference problems that arise when the same data are used to both define and test predictors [15]. Previous studies reporting single-region predictions [45, 60] often derived predictors post-hoc, likely inflating effect sizes and limiting external validity.

A limitation is that our study was a secondary analysis, pooling participants from a treatment trial. This is unlikely to be a major limitation, however, as the rTMS treatment used failed to influence any of the outcomes measured, even at a trend level [21]. Nevertheless, the small sample size of 39 participants, including the underrepresentation of female subjects, may limit the generalizability and bias the outcomes, as well as increase the risk of chance findings in larger numbers of analyses. Also, the use of a median split in the survival analysis necessarily discards information and may impact the effect sizes. However, the improved predictive power of the median split model over a model using NCS scores as a continuous predictor is of potential interest, because it indicates that the contribution of NCS to relapse risk is non-linear, and that a threshold value likely exists above which risk steeply rises. Finally, while the convergence across multiple outcomes supports their validity, we did not formally adjust for multiplicity across these, and thus secondary associations should be interpreted as tentative. Taken together, a need remains for replicating these findings in prospective studies, and future trials should consider collecting cue reactivity data at the start of treatment so that subsequent outcomes (e.g., treatment dropout) may be analyzed as a function of NCS score. Future research should examine whether the NCS is a dynamic marker that is responsive to treatment, and measures its efficacy. For example, one recent study suggests NCS responsiveness to dietary treatment [20].

The NCS was developed to predict in-session craving and was also found to distinguish individuals with vs. without SUDs using data from a specific “regulation of craving” task [19]. Two key questions were left open by that foundational study. First, multiple cue provocation paradigms are in use in the field [5]. An important question was therefore whether the NCS generalizes to other commonly used paradigms. Our data, obtained using a very different procedure to evoke brain responses to alcohol associated cues, support this generalizability. Interestingly, the NCS appears to have captured craving-related brain responses, despite the potential differences in cognitive demands between this stimulus identification task relative to the “regulation of craving” task. Future studies should explore generalizability across additional craving-induction methods, such as contextual cues, movies, priming doses, or stress, and test generalizability across levels of cognitive load. Still, our initial results suggest that cognitive load of the task does not result in any inefficiencies in the NCS’ predictive power. Second, while the original study validated the NCS as a neural marker of craving, and as a classifier of people with and without an SUDs, it left open the question of whether it would also discriminate high vs. low severity or prospectively predict clinical outcomes. Despite the limitations above, our findings strongly support both these notions. Importantly, the NCS demonstrated robust predictive associations with clinical outcomes in AUD using a straightforward fMRI protocol, that can easily be implemented both in research and clinical settings.

Collectively, these initial findings provide initial support for the NCS as a prognostic neuromarker for relapse in AUD, and a rationale for future studies to assess the utility of the NCS as a neuromarker predictive of outcomes and treatment responses, potentially advancing personalized approaches to AUD treatment.

## Supplementary information

Supplemental Material

## Data Availability

Data available on request, pending requisite Swedish Ethics Authority review.
