# Supplementary material for: The neurobiological cravings signature (NCS) as a predictive neuromarker of clinical outcomes in alcohol use disorder
Source: Neuropsychopharmacology. 2026 Feb 25;51(7):1237–44. doi: 10.1038/s41386-026-02369-3 (PMC13212722; doi:10.1038/s41386-026-02369-3)
Supplement: Supplementary file 1 — Supplemental Material [file 41386_2026_2369_MOESM1_ESM.docx]

**Supplementary Materials**

The Neurobiological Cravings Signature (NCS) as a Predictive Neuromarker of Clinical Outcomes in Alcohol Use Disorder

Andreas Löfberg*, MD,^1,2^ Nicholas Harp*, PhD,^3^ Irene Perini, PhD,^1^ Robin Kämpe, MSc,^1^ Hanna Karlsson, MD, PhD,^1,2^ Michal Pietrzak MD,^1,2^ Hedy Kober*, PhD,^3^ Markus Heilig*, MD, PhD^1,2^

1. Center for Social and Affective Neuroscience, Department of Biomedical and Clinical Sciences, Linköping University, Linköping, Sweden.

2. Department of Psychiatry, Linköping University Hospital, Linköping, Sweden.

3. Department of Psychology, University of California Berkely, California, USA.

**PARTICIPANT DISPOSITION**

**
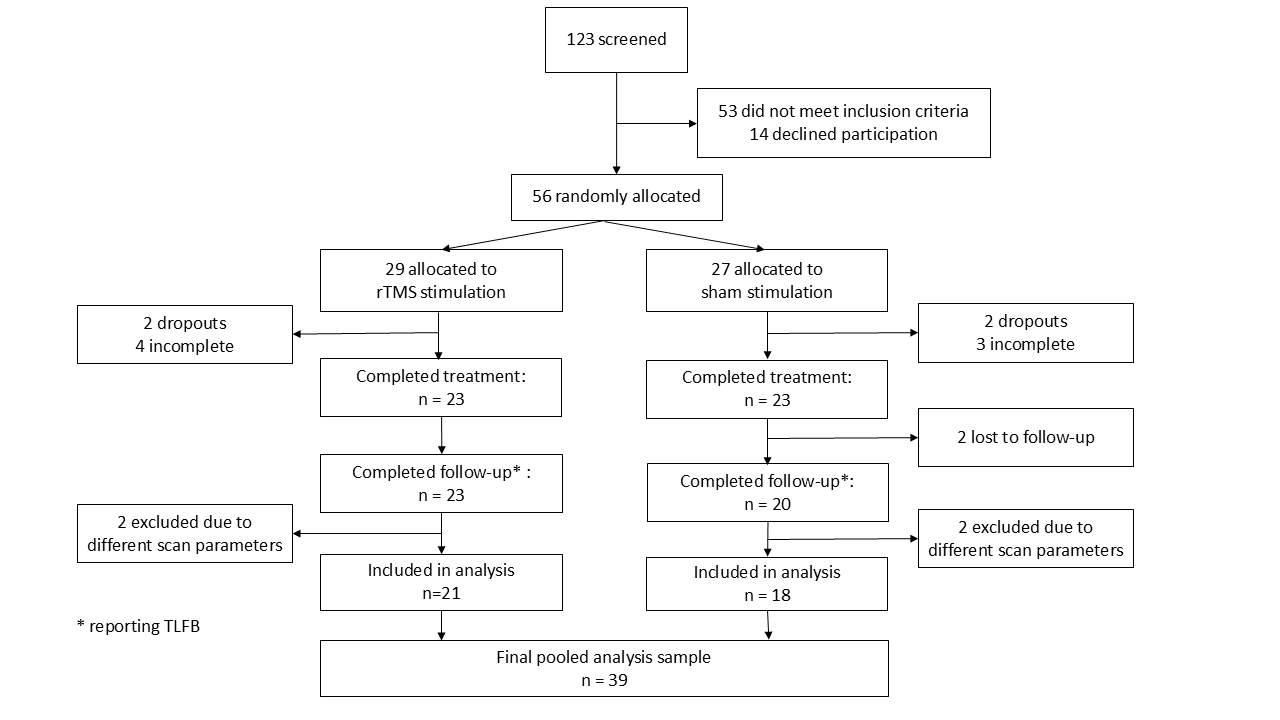
**

**Figure S1**. CONSORT-graph of participant in the current secondary analysis of the original rTMS RCT.

**SUPPLEMENTARY RESULTS**

*Dichotomization versus continuous modeling of NCS in relapse prediction*Although dichotomization can reduce statistical power, in this dataset, the median split performed better than the continuous analysis. This suggests is a nonlinear risk function, in which relapse risk increases sharply among individuals with higher NCS expression. A linear Cox model dilutes this threshold-like relationship, whereas the binary contrast efficiently captures it. We also note that multivariate neural signatures can exhibit heteroscedastic measurement error, which disproportionately attenuates continuous predictors but has less impact on group-based contrasts. These factors likely explain why the dichotomous analysis showed stronger predictive performance here.

*
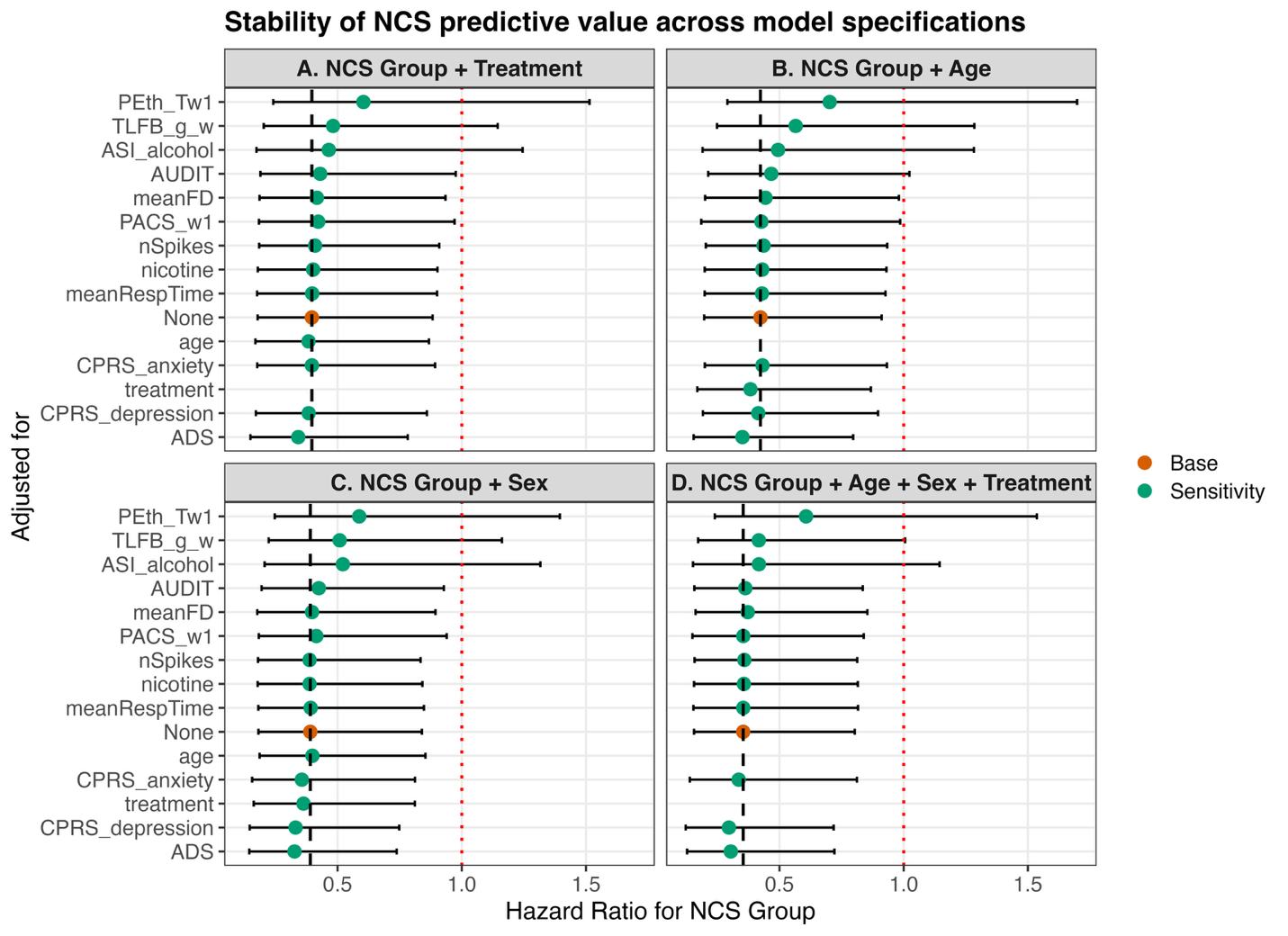
*

**Figure S2.** Sensitivity analyses of relapse prediction by NCS group (median split).

**
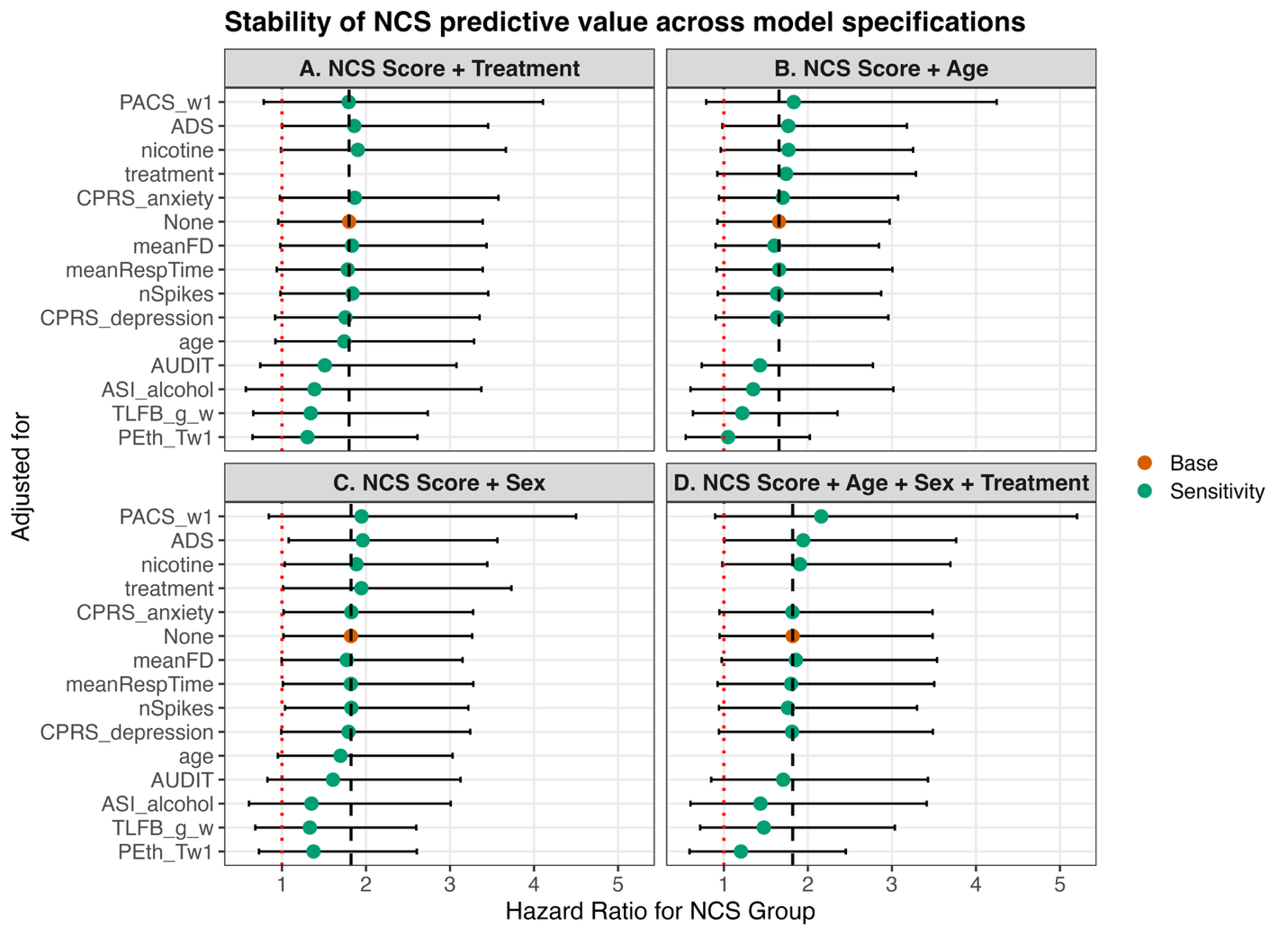
**

**Figure S3.** Sensitivity analyses of relapse prediction by NCS score (continuous).

*Bayesian Weibull survival model*

The Bayesian Weibull model yielded a posterior estimate for the intercept of 5.15 (95% credible interval [2.98, 8.35]). The coefficient for Low NCS group was –1.38 (95% CI [–2.25, –0.59], **Figure S1**), indicating that low NCS is associated with a significantly lower hazard of relapse (median hazard ratio = exp(–1.38) ≈ 0.25). The treatment effect was not significant (estimate 0.24, 95% CI [–0.66, 1.04]), while age showed a slight negative association (–0.04, 95% CI [–0.09, 0.00]). Males had a higher risk of relapse (estimate 1.22, 95% CI [0.63, 1.82]). The Weibull shape parameter was estimated at 2.10 (95% CI [1.10, 3.75]), suggesting a hazard that changes over time. Convergence diagnostics indicated reliable estimates (all Rhat values ≈ 1.00 with robust effective sample sizes). Posterior predictive checks confirmed that the model adequately captured the observed survival distribution.


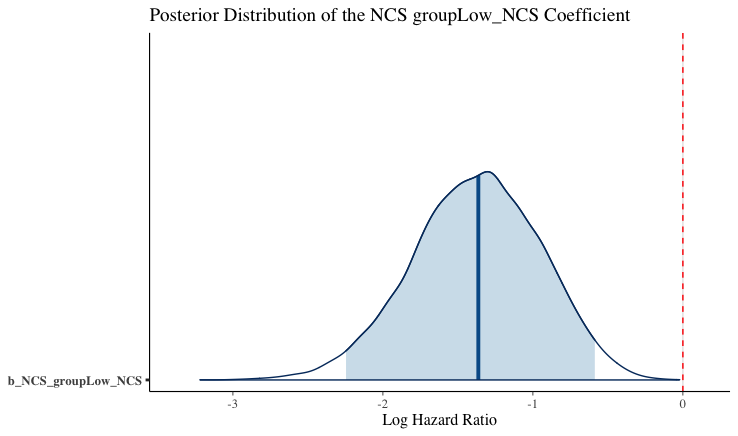


**Figure S4**. Distribution estimates of posterior MCMC distribution of the Low NCS group on survival to relapse compared to the High NCS group. Median Log HR (-1.38) demarked by thick blue line. Shaded blue area is 95% credibility interval, all below 0, indicating high significance.

*Bayesian Cox Model*

The Bayesian Cox model indicated a credible negative effect on relapse hazard for the low NCS group (β = -1.14, 95% CI = [-1.96, -0.34]). Sex (male) was associated with an increased hazard rate (β = 1.12, 95% CI = [0.07, 2.30]), while age and treatment showed smaller effects with credible intervals including zero.  Bayesian model comparison using bridge sampling resulted in a Bayes factor (BF) of 4.08, favoring the full model (including NCS group) over the null model (excluding NCS group). This indicates moderate evidence supporting the inclusion of NCS group in the model.


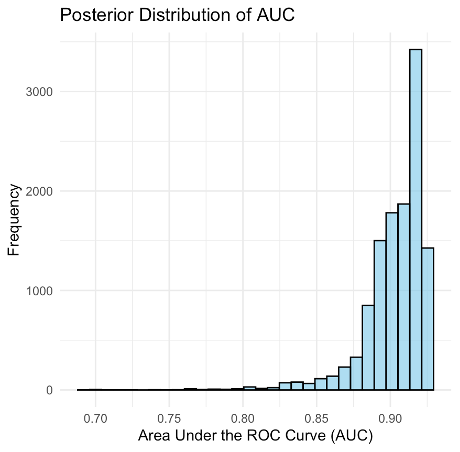


**Figure S5**. Distribution of posterior estimates of AUC values from the Bayesian ROC approach.

*Sensitivity Analyses*

Below we show sensitivity analyses of the PEth, PACS, and heavy drinking prediction when accounting for various clinical and methodological covariates (**Figure S6**), as well as associations among NCS and key covariates (**Figure S7**).


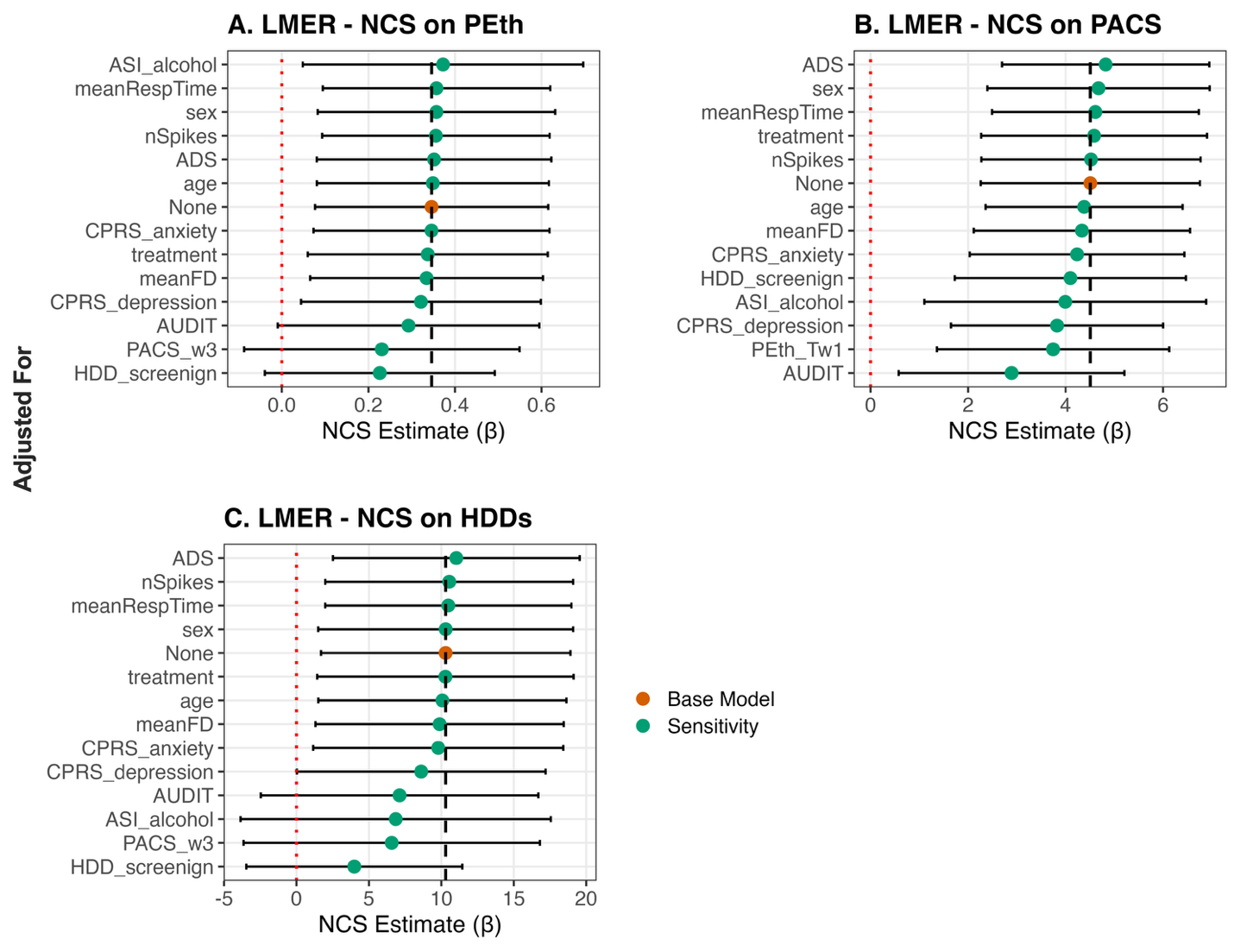


**Figure S6.** Sensitivity analyses for PEth, PACS, and heavy drinking.


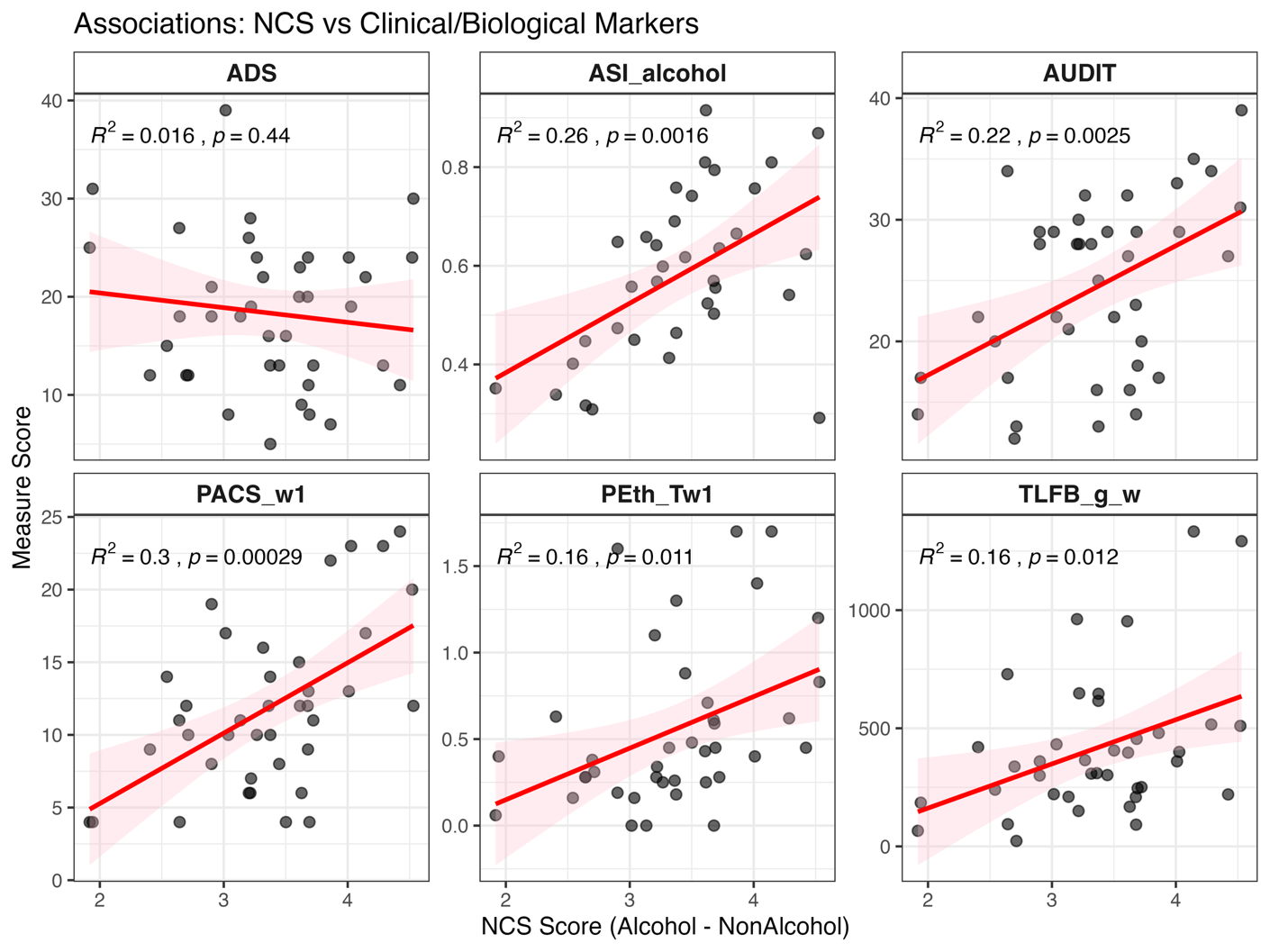


**Figure S7.** Spearman correlations for NCS and key covariates.

*Post hoc power analyses*

We ran post-hoc power analyses for PEth and relapse prediction using the simr package for R. For the main outcome, the linear mixed effect model with NCS predicting PEth achieved 75% power (95% CI 72-78) to detect the effect size (beta 0.36), slightly below the conventional 80% threshold. Future studies should target >45 subjects using this method. Similarly, a *post hoc* power analysis, using the powerSurvEpi package for R, showed that the Cox regression achieved 74% power, and that future studies should similarly use sample sizes of >45.


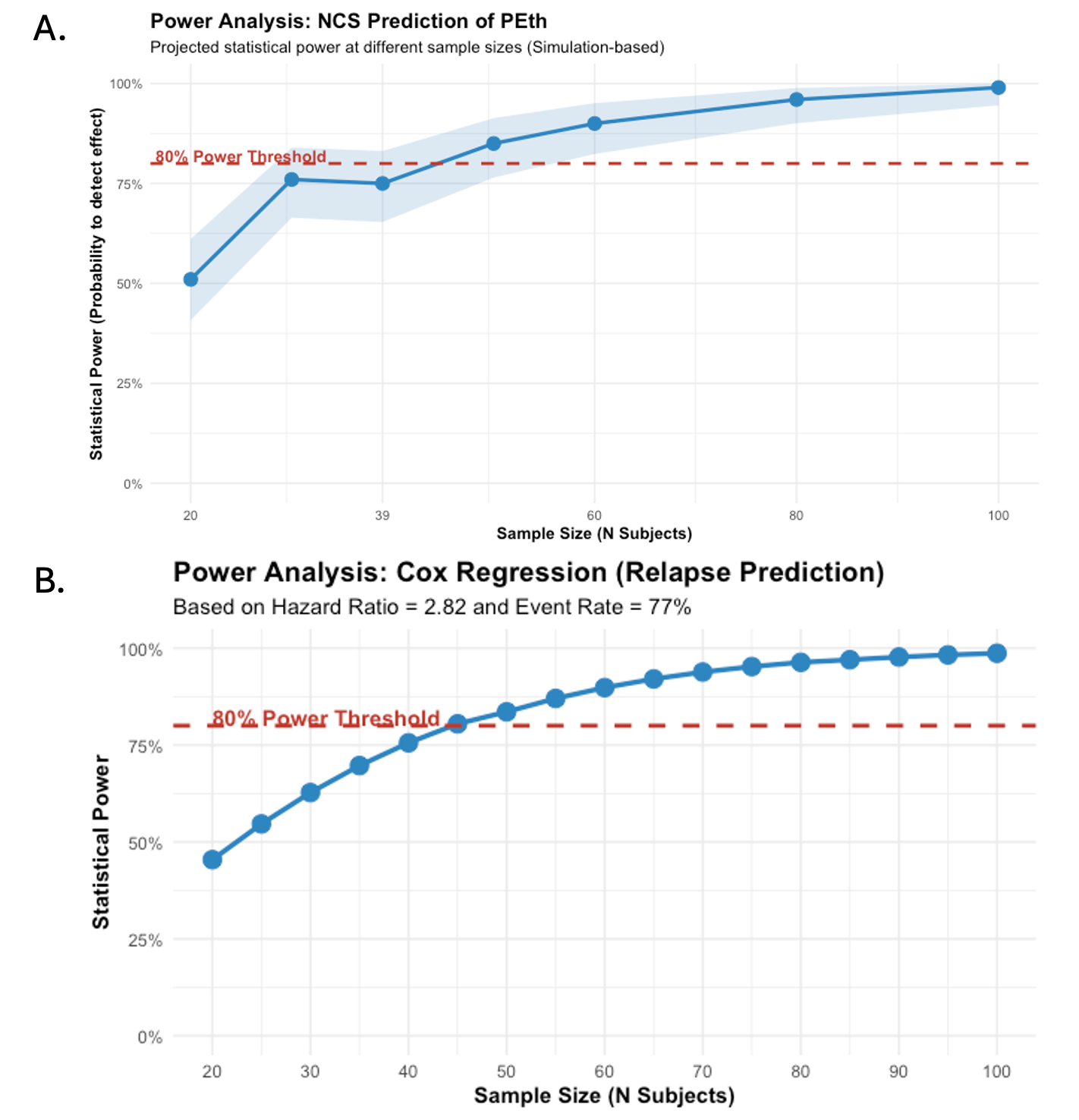


**Figure S8.** Post hoc power analysis for NCS prediction of PEth and relapse.

| **NCS_group** | **domain** | **Week 1** | **Week 2** | **Week 4** | **Week 8** | **Week 12** |
| --- | --- | --- | --- | --- | --- | --- |
| **Low NCS** | **Alcohol use (TLFB)** | 0 (0%) | 1 (5.3%) | 1 (5.3%) | 0 (0%) | 0 (0%) |
|  | **Craving (PACS)** | 3 (15.8%) | 2 (10.5%) | 2 (10.5%) | 0 (0%) | 0 (0%) |
|  | **Heavy drinking days (HDD)** | 0 (0%) | 0 (0%) | 1 (5.3%) | 0 (0%) | 0 (0%) |
| **High NCS** | **Alcohol use (TLFB)** | 1 (5%) | 1 (5%) | 4 (20%) | 1 (5%) | 1 (5%) |
|  | **Craving (PACS)** | 1 (5%) | 2 (10%) | 3 (15%) | 1 (5%) | 3 (15%) |
|  | **Heavy drinking days (HDD)** | 2 (10%) | 1 (5%) | 3 (15%) | 1 (5%) | 2 (10%) |

**Table S1. Missingness across longitudinal outcomes stratified by NCS group.** Cells indicate the number (%) of missing observations at each follow-up timepoint for craving (PACS), alcohol use (TLFB), and heavy drinking days (HDD).
